# Functional Connectivity Profiles of Ten Sub-Regions within the Premotor and Supplementary Motor Areas: Insights into Neurophysiological Integration

**DOI:** 10.3390/diagnostics14171990

**Published:** 2024-09-09

**Authors:** Adnan Alahmadi

**Affiliations:** Radiologic Sciences, Faculty of Applied Medical Sciences, King Abdulaziz University, Jeddah, Saudi Arabia; aaalahmadi@kau.edu.sa

**Keywords:** motor, functional connectivity, resting-state fMRI, premotor cortex, supplementary motor area, brain networks

## Abstract

**Objectives:** This study aimed to comprehensively investigate the functional connectivity of ten sub-regions within the premotor and supplementary motor areas (Right and Left Premotor 6d1, 6d2, 6d3, and Right and Left pre-Supplementary Motor (presma) and SMA). Using advanced magnetic resonance imaging (MRI), the objective was to understand the neurophysiological integrative characteristics of these regions by examining their connectivity with eight distinct functional brain networks. While previous studies have largely treated these areas as homogeneous entities, there is a significant gap in our understanding of the specific roles and connectivity profiles of their distinct sub-regions. The goal was to uncover the roles of these regions beyond conventional motor functions, contributing to a more holistic understanding of brain functioning. **Methods:** The study involved 198 healthy volunteers, with the primary methodology being functional connectivity analysis using advanced MRI techniques. Ten sub-regions within the premotor and supplementary motor areas served as seed regions, and their connectivity with eight distinct brain regional functional networks, including the Sensorimotor, Dorsal Attention, Language, Frontoparietal, Default Mode, Cerebellar, Visual, and Salience networks, was investigated. This approach allowed for the exploration of synchronized activity between these critical brain areas, shedding light on their integrated functioning and relationships with other brain networks. **Results:** The study revealed a nuanced landscape of functional connectivity for the premotor and supplementary motor areas with the main functional brain networks. Despite their high functional connectedness within the motor network, these regions displayed diverse functional integrations with other networks. There was moderate connectivity with the Sensorimotor and Dorsal Attention networks, highlighting their roles in motor execution and attentional processes. However, connectivity with the Language, Frontoparietal, Default Mode, Cerebellar, Visual, and Salience networks was generally low, indicating a primary focus on motor-related tasks. **Conclusions:** This study emphasized the multifaceted roles of the sub-regions of the premotor and supplementary motor areas. Beyond their crucial involvement in motor functions, these regions exhibited varied functional integrations with different brain networks. The observed disparities, especially in the Sensorimotor and Dorsal Attention networks, indicated a nuanced and specialized involvement of these regions in diverse cognitive functions. By delineating the specific connectivity profiles of these sub-regions, this study addresses the existing knowledge gap and suggests unique and distinct roles for each brain area in sophisticated cognitive tasks beyond their conventional motor functions. The results suggested unique and distinct roles for each brain area in sophisticated cognitive tasks beyond their conventional motor functions. This study underscores the importance of considering the broader neurophysiological landscape to comprehend the intricate roles of these brain areas, contributing to ongoing efforts in unravelling the complexities of brain function.

## 1. Introduction

The premotor and supplementary motor areas are integral to a wide range of motor functions, encompassing the planning, coordination, and execution of movements [1]. These regions are not limited to mere motor control; they play crucial roles in complex neurological functions. Previous studies have highlighted their involvement in various higher-order cognitive and attentional processes. For instance, some studies demonstrated that these areas are critical for the initiation and sequencing of movements, which are essential for performing coordinated actions [1]. For instance, the role of the premotor area in learning and executing sequential tasks has been shown alongside its importance in motor learning and adaptation [2,3,4,5]. The supplementary motor area, on the other hand, has been associated with internally generated movements and the coordination of bimanual activities, as shown in various neuroimaging studies [2,6,7,8]. Recent research has expanded our understanding of these areas, illustrating their involvement in attentional processes and higher-order cognitive tasks. This includes functions such as decision-making, error detection, and the integration of sensory information to guide motor actions [2]. The intricate connectivity and interaction between the premotor and supplementary motor areas with other brain regions underscore their multifaceted roles in both motor and cognitive domains.

Resting-state fMRI (rsfMRI) offers a powerful tool to explore the brain’s functional network dynamics by examining spontaneous low-frequency fluctuations during rest. These fluctuations are structurally correlated and can reveal functional networks that are consistently involved in both resting and active states [9]. The premotor area is intricately involved in the planning and execution of movements, spatial guidance, and the understanding of others’ actions. Key studies have demonstrated that the premotor area communicates with several brain regions to perform these functions. For instance, the premotor area is crucial for motor planning, working closely with the primary motor cortex (M1) and the parietal cortex to translate sensory information into motor commands. This connection is essential for preparing and planning movements [10]. Additionally, the premotor area integrates sensory information through its strong connectivity with the posterior parietal cortex, which guides motor actions [11]. The ventral part of the premotor area is also part of the mirror neuron system, connecting to the inferior frontal gyrus to facilitate the understanding and imitation of actions [12].

The supplementary motor area (SMA) plays a significant role in initiating movement, bimanual coordination, and sequential movements. Its connectivity with various brain regions supports these functions. The SMA’s strong connectivity with the basal ganglia and prefrontal cortex facilitates the learning and execution of motor sequences, highlighting its role in motor sequence learning [1]. Furthermore, the SMA’s connection with the contralateral SMA and M1 is vital for coordinating movements that involve both hands, emphasizing its importance in bimanual coordination [1]. The pre-SMA, a part of the SMA, is connected to the anterior cingulate cortex and the prefrontal cortex, playing a crucial role in the initiation and inhibition of movements [13].

Traditionally, these regions have been treated as homogeneous entities in functional magnetic resonance imaging (fMRI) studies. However, recent cytoarchitectonic analyses reveal a complex, heterogeneous structure within these areas, composed of several distinct sub-regions [14,15,16]. These include the Right and Left Premotor 6d1, 6d2, 6d3, and Right and Left pre-presma and SMA. These advancements in cytoarchitectonic mapping, have provided probabilistic maps and detailed analyses of these regions. These maps enable the identification of distinct sub-regions with unique cytoarchitectonic properties and receptor distribution patterns.

Despite these advancements, there remains a significant gap in the literature regarding the specific functional connectivity profiles of these sub-regions within the premotor and SMA. Most studies have treated these areas as homogeneous, overlooking the nuanced connectivity and distinct functional roles of their sub-regions. This lack of a detailed understanding limits our comprehension of their broader neurophysiological roles.

The primary objective of this study is to investigate the functional connectivity of ten sub-regions within the premotor and SMA. These sub-regions are hypothesized to exhibit distinct functional connectivity profiles with major brain networks, including the sensorimotor, dorsal attention, language, frontoparietal, default mode, cerebellar, visual, and salience networks, due to their anatomical and cytoarchitectonic differences. By examining these connections, we aim to uncover the roles of these sub-regions beyond conventional motor functions and anatomical distinctions, contributing to a more comprehensive understanding of brain functioning. This study aims to fill the existing knowledge gap by providing detailed insights into the specific functional connectivity of these sub-regions, thereby enhancing our understanding of their roles in both motor and cognitive processes.

## 2. Methods

### 2.1. Subject Recruitment

This study involved a cohort of 198 healthy volunteers aged between 18 and 30 years. The average age of the participants was 21.03 years, with a standard deviation of 2.31 years. The sample consisted of 123 females and 75 males (Table 1), ensuring a balanced representation of genders. To assess handedness, we used the Edinburgh Handedness Inventory, a widely recognized tool for determining hand preference. The results showed that 171 participants were right-handed, reflecting the general population distribution. Importantly, all participants were screened to confirm the absence of any neurological symptoms or disorders, ensuring the integrity of the study. The data were sourced from the Cambridge–Buckner dataset, which is part of the 1000 Functional Connectomes Project, an open access platform providing a valuable resource for neuroscience research [9]. The Institutional Review Board (IRB) statement for this project is available at [IRP statement] (http://fcon1000.projects.nitrc.org) (accessed on 22 April 2024), ensuring ethical compliance and participant consent.

### 2.2. Scanning

Imaging was conducted using a Siemens 3 Tesla Trim Trio scanner, a high-field MRI system that provides excellent image quality and resolution. Resting-state functional MRI (rsfMRI) scans were performed using a T2* weighted Echo Planar Imaging (EPI) sequence, selected for its sensitivity to blood oxygen level-dependent (BOLD) contrasts. The imaging parameters were set as follows: a repetition time (TR) of 3000 milliseconds, an echo time (TE) of 30 milliseconds, and 47 interleaved axial slices that covered the entire brain, including the cerebellum. Each voxel measured 3.0 × 3.0 × 3.0 mm^3^, providing a high level of detail across the 119 volumes acquired per participant. Additionally, T1-weighted anatomical images were captured with 192 slices, a 144 × 192 matrix, and a voxel size of 1.20 × 1.00 × 1.33 mm^3^, facilitating precise anatomical localization and normalization of the functional data.

### 2.3. Pre-Processing

The rsfMRI data underwent comprehensive pre-processing using a combination of Statistical Parametric Mapping software (SPM12), the CONN toolbox, and MATLAB (R2020b). Pre-processing steps were meticulously carried out to ensure data quality and accuracy. Initially, slice timing corrections were applied to adjust for differences in acquisition time between slices. Next, the functional volumes were realigned to correct for head movements, followed by normalization to a common space template using the structural data of each subject. This step ensures that the functional data are comparable across subjects. Finally, the functional volumes were smoothed with an 8 mm^3^ Gaussian kernel to enhance signal-to-noise ratio and prepare the data for statistical analysis.

### 2.4. Selection of Regions of Interest (ROIs)

Regions of interest (ROIs) were meticulously selected to ensure precision in the analysis. The ten regions were delineated using the Cytoarchitectonics Julich-Brain Atlas, available in the Julich-Brain toolbox. The selected ROIs include Right Premotor 6d1, Left Premotor 6d1, Right Premotor 6d2, Left Premotor 6d2, Right Premotor 6d3, Left Premotor 6d3, Right presma, Left presma, Right SMA, and Left SMA. In addition to these specific regions, we identified target brain areas as extensive clusters within functional networks provided in the CONN toolbox. These networks, characterized by distinct features, included the default mode network, sensorimotor network, visual network, salience network, dorsal attention network, frontoparietal network, cerebellar network, and language network. This comprehensive selection of ROIs and networks ensures a robust analysis of functional connectivity across various brain regions.

### 2.5. Statistical Analysis

Statistical analyses were performed at both the subject and group levels. At the subject level, weighted general linear bivariate correlation models were employed to construct matrices of ROI-to-ROI links for each participant. These models utilized bivariate Fisher-transformed correlation coefficients between paired ROI timeseries, allowing for a detailed examination of functional connectivity. At the group level, functional connectivity measurements were computed and compared using T-tests and F-tests. This analysis identified and compared rsfMRI networks associated with each of the cerebellar regions, providing insights into the connectivity patterns of these areas. To ensure statistical robustness, a corrected false discovery rate (FDR) of *p* < 0.05 was applied using a multivariate parametric general linear model analysis. This correction accounts for multiple comparisons, reducing the likelihood of Type I errors and ensuring the reliability of the results.

## 3. Results

**Figure 1:**
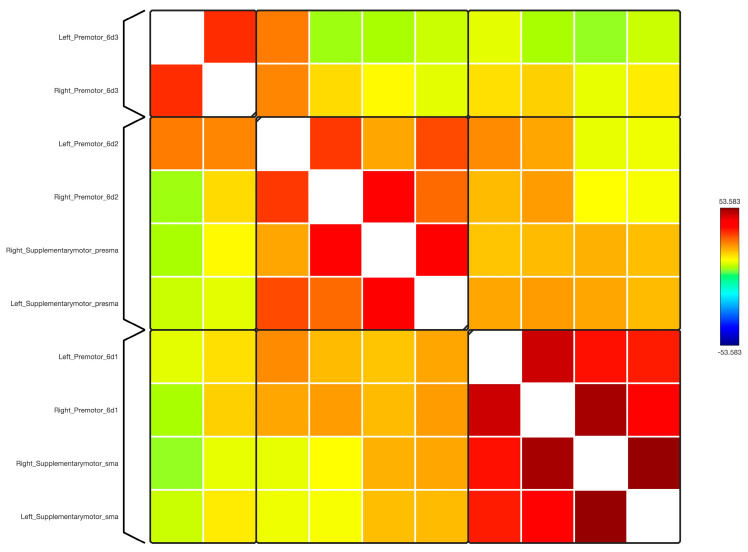
The functional connectivity in a matrix diagram of the 10 sub-regions of the premotor and SMA is shown at the group level. This figure shows statistical specializations of the selected seed functional connectivity. The colours of the matrix are proportional to statistical strength, and the T-bar is shown at the top right-hand corner.

**Figure 2:**
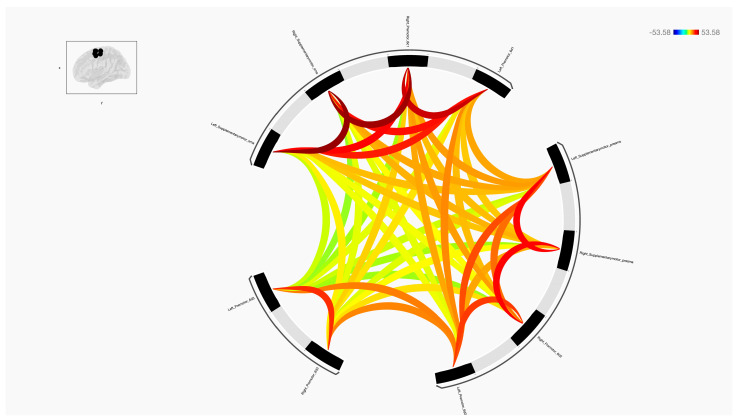
The functional connectivity in a ring diagram of the 10 sub-regions of the premotor and SMA is shown at the group level. This figure shows statistical specializations of the selected seed functional connectivity. The colours of the lines are proportional to statistical strength, and the T-bar is shown at the top right-hand corner.

**Figure 3:**
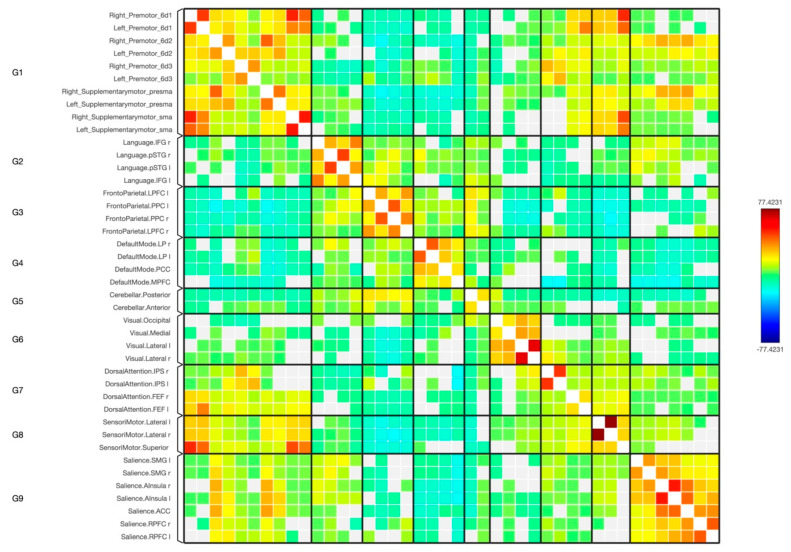
The functional connectivity in a matrix diagram of the 10 sub-regions of the premotor and SMA with the targeted functional brain networks is shown at the group level. This figure shows statistical specializations of the selected seed functional connectivity with the target networks. The colours of the matrix are proportional to statistical strength, and the T-bar is shown at the top right-hand corner.

**Figure 4:**
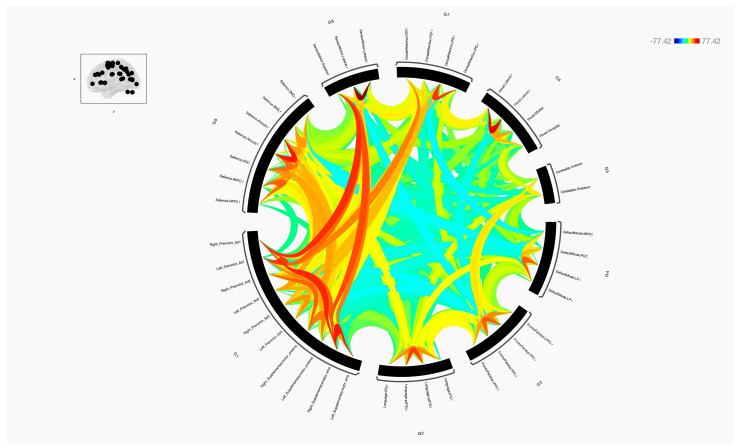
The functional connectivity in a wring diagram of the 10 sub-regions of the premotor and SMA with the targeted functional brain networks is shown at the group level. This figure shows statistical specializations of the selected seed functional connectivity with the target networks. The colours of the lines are proportional to statistical strength, and the T-bar is shown at the top right-hand corner.

In this study, a detailed analysis of the functional connectivity strength of eight sub-regions within the premotor and supplementary motor areas was conducted and these were compared with regions from across the major brain functional networks, including the Language, Frontoparietal, Default Mode, Cerebellar, Visual, Dorsal Attention, Sensorimotor, and Salience networks (Figure 1, Figure 2, Figure 3 and Figure 4).

### 3.1. Right Premotor 6d1 (Region 1)

The Right Premotor 6d1 region displayed robust intranetwork connectivity within the premotor and supplementary motor areas. It showed particularly strong connectivity with Left Premotor 6d1, Right Supplementary Motor (presma), and Left Supplementary Motor (presma). Connectivity with other regions such as the Language network (Language.IFG r, Language.pSTG l), Frontoparietal network (FrontoParietal.PPC r, FrontoParietal.PPC l), Default Mode network (DefaultMode.PCC, DefaultMode.MPFC), Cerebellar network (Cerebellar.Anterior, Cerebellar.Posterior), Visual network (Visual.Occipital, Visual.Lateral l), Dorsal Attention network (DorsalAttention.IPS r, DorsalAttention.FEF r), Sensorimotor network (Sensorimotor.Lateral r, Sensorimotor.Superior), and Salience network (Salience.ACC, Salience.RPFC r) was generally moderate to low. The highest connectivity was observed with the Sensorimotor and Dorsal Attention regions.

### 3.2. Left Premotor 6d1 (Region 2)

The Left Premotor 6d1 region exhibited strong connectivity within the premotor and supplementary motor areas, similar to Right Premotor 6d1. It showed strong connections with Right Premotor 6d1 and other regions within this motor network. Its connectivity with other networks, particularly the Frontoparietal network (FrontoParietal.LPFC l, FrontoParietal.PPC l), was slightly higher compared to Right Premotor 6d1. Connectivity with other networks such as the Language, Default Mode, Cerebellar, Visual, Dorsal Attention, Sensorimotor, and Salience networks remained moderate to low.

### 3.3. Right Premotor 6d2 (Region 3)

The Right Premotor 6d2 region maintained high connectivity within the premotor and supplementary motor areas, especially with Right Premotor 6d1 and Right Premotor 6d3. It demonstrated moderate connectivity with the Sensorimotor (Sensorimotor.Lateral r) and Dorsal Attention (DorsalAttention.IPS r) networks. A higher connection was observed with most of the Salience networks. Connectivity with the Language, Frontoparietal, Default Mode, Cerebellar, and Visual was lower.

### 3.4. Left Premotor 6d2 (Region 4)

The Left Premotor 6d2 region showed strong connectivity within the premotor and supplementary motor areas, particularly with Left Premotor 6d1 and Left Premotor 6d3. It exhibited moderate connectivity with the Frontoparietal (FrontoParietal.PPC l) and Sensorimotor (Sensorimotor.Lateral l) networks. Connectivity with other networks such as the Language, Default Mode, Cerebellar, Visual, Dorsal Attention, and Salience networks was generally low.

### 3.5. Right Premotor 6d3 (Region 5)

The Right Premotor 6d3 region demonstrated a very strong intranetwork connectivity within the premotor and supplementary motor areas, especially with Right Premotor 6d2. Its connectivity with other networks was moderate to low, with higher interactions observed with the Sensorimotor (Sensorimotor.Lateral r) network. A higher connection was observed with most of the dorsal attention networks. The connectivity with the Language, Frontoparietal, Default Mode, Cerebellar, Visual, Dorsal Attention, and Salience networks was lower.

### 3.6. Left Premotor 6d3 (Region 6)

The Left Premotor 6d3 region showed a strong connectivity with the premotor and supplementary motor areas, particularly with the Left Premotor 6d2. Similar to Right Premotor 6d3, it exhibited a moderate to low connectivity with other networks, with a notable interaction with the Sensorimotor (Sensorimotor.Lateral l) network. A higher connection was observed with most of the dorsal attention networks. Its connectivity with the Language, Frontoparietal, Default Mode, Cerebellar, Visual, and Salience networks was generally low.

### 3.7. Right Supplementary Motor (Presma) (Region 7)

The Right Supplementary Motor (presma) region maintained a strong connectivity with the premotor and supplementary motor areas, especially with right-side regions such as the Right Premotor 6d1. It demonstrated a moderate connectivity with the Frontoparietal (FrontoParietal.PPC r) and Dorsal Attention (DorsalAttention.FEF r) networks. A higher connection was observed with most of the Salience networks. Its connectivity with the Language, Default Mode, Cerebellar, Visual, and Sensorimotor was generally low.

### 3.8. Left Supplementary Motor (Presma) (Region 8)

The Left Supplementary Motor (presma) region exhibited a strong connectivity with left-side Premotor regions and other regions within the premotor and supplementary motor areas. It showed a moderate connectivity with the Frontoparietal (FrontoParietal.PPC l) and Sensorimotor (Sensorimotor.Lateral l) networks. Its connectivity with the Language, Default Mode, Cerebellar, Visual, Dorsal Attention, and Salience networks was generally low.

### 3.9. Right Supplementary Motor (SMA) (Region 9)

The Right Supplementary Motor (SMA) region maintained a positive connectivity with the premotor and supplementary motor areas, especially with right-side regions such as the Right Premotor 6d1 and Left SMA. It demonstrated a high connectivity with sensorimotor network. A moderate connection was observed with most of the Salience networks. Its connectivity with the Language, Default Mode, Cerebellar, Visual, and Sensorimotor was generally low.

### 3.10. Left Supplementary Motor (SMA) (Region 10)

**Figure 5:**
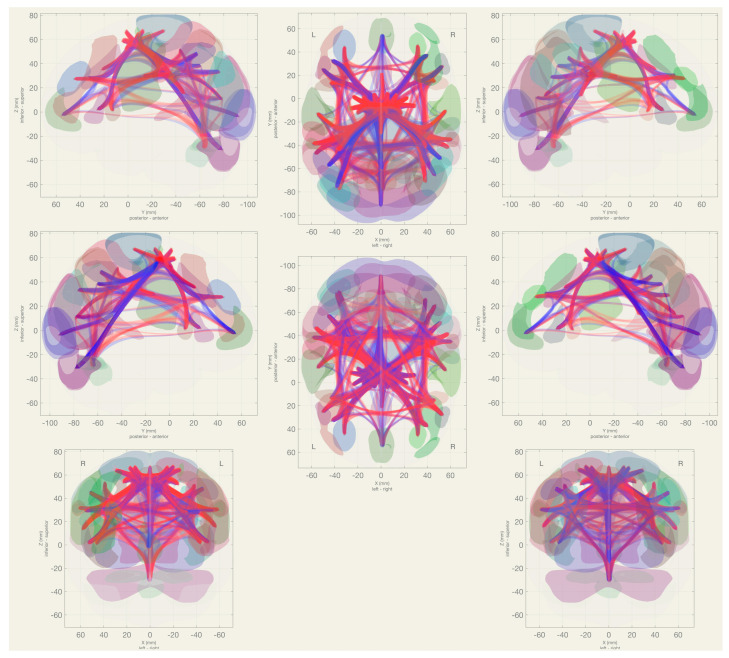
The functional MRI connectivity of the 10 sub-regions of the premotor and SMA is shown at the group level and presented over an inflated surface for the purpose of snowing.

The Left Supplementary Motor (SMA) region maintained a positive connectivity with the premotor and supplementary motor areas, especially with right-side regions such as the Right Premotor 6d1 and Left SMA. It demonstrated a high connectivity with sensorimotor network. A moderate connection was observed with most of the Salience networks. Its connectivity with the Language, Default Mode, Cerebellar, Visual, and Sensorimotor was generally low (Figure 5).

## 4. Discussion

The aim of this study was to comprehensively investigate the functional connectivity profiles of ten sub-regions within the premotor and supplementary motor areas (Right and Left Premotor 6d1, 6d2, 6d3, and Right and Left presma and SMA) to understand their neurophysiological integrative characteristics beyond conventional motor functions. Using advanced resting-state functional magnetic resonance imaging (rsfMRI), this study examined the connectivity of these regions with eight distinct functional brain networks: the Sensorimotor, Dorsal Attention, Language, Frontoparietal, Default Mode, Cerebellar, Visual, and Salience networks. The analysis involved a large cohort of 198 healthy volunteers, and the functional connectivity was assessed using ROI-ROI-based analysis within and between these sub-regions and the targeted networks. The premotor cortex and supplementary motor areas (SMAs) exhibited distinct anatomical features across their sub-regions [14,15,16]. The premotor cortex is divided into areas 6d1, 6d2, and 6d3. Premotor 6d1 is situated in the superior part of the premotor cortex, and is characterized by a homogeneous cytoarchitectonic structure and strong connections with the primary motor cortex and parietal cortex, facilitating motor planning and spatial attention. Premotor 6d2, located more ventrally, shows distinct laminar differentiation and is involved in fine-tuning motor actions. Premotor 6d3, found in the most ventral part of the superior premotor cortex, has robust intranetwork connectivity within the premotor and supplementary motor areas, emphasizing its role in integrating sensory inputs for motor tasks. The SMA and pre-SMA also differ anatomically. The SMA, located on the medial surface of the hemisphere anterior to the primary motor cortex, is characterized by poor lamination and densely packed large pyramidal cells, with strong corticospinal projections. It is crucial for motor sequence learning and bimanual coordination. The pre-SMA, positioned anterior to the SMA, exhibits a more differentiated laminar pattern with a pronounced layer V and lacks direct connections to the primary motor cortex. Instead, it receives inputs from the anterior premotor cortex, midfrontal cortex, and midcingulate area 24c, playing a role in complex movement patterns and motor inhibition.

The Right and Left Premotor regions (6d1, 6d2, 6d3), the presma, and SMA exhibited robust intranetwork connectivity, which is crucial for coordinated motor planning and execution. This strong internal connectivity aligns with previous studies, emphasizing the integrated role these regions play in facilitating complex motor tasks [1]. The connectivity of the sub-regions of the premotor and supplementary motor areas with other brain networks varied, reflecting their functional specialization [17,18].

The connectivity of the right premotor area 6d1 with the sensorimotor network underscores its role in integrating sensory input with motor planning and execution, as supported by various studies [17,18,19,20]. Additionally, the connectivity of this sub-region with the dorsal attention network suggests its involvement in spatial attention and the coordination of visually guided movements [19,20]. Research has shown that the functional network connectivity between the DAN and other networks, including the sensorimotor network, plays a critical role in attention and motor functions, further supporting the idea that premotor area 6d1 contributes to these processes [19,20,21]. The low connectivity with the Language network indicates limited involvement in language processing. Moderate connectivity with the Frontoparietal network implies a role in higher-order executive functions and cognitive control [22]. The generally low connectivity with the Default Mode, Cerebellar, Visual, and Salience networks highlights its primary focus on motor-related tasks rather than cognitive or sensory processes. The results showed that the Left Premotor 6d1 exhibited similar connectivity patterns to the Right Premotor 6d1, with a strong connectivity with the Sensorimotor network, emphasizing its role in motor control. Moderate connectivity with the Dorsal Attention network reflects its involvement in attention-related motor tasks. The slightly higher connectivity with the Frontoparietal network compared to Right Premotor 6d1 could suggest potential lateralized functions in executive control. The low connectivity with the Default Mode, Cerebellar, Visual, and Salience networks reinforces its motor-centric functions.

The Right Premotor 6d2 maintained high connectivity within the premotor and supplementary motor areas, particularly with Right Premotor 6d1 and Right Premotor 6d3. It displayed moderate connectivity with the Sensorimotor and Dorsal Attention networks, aligning with its role in fine-tuning motor actions and directing attention to motor tasks [21,23,24,25]. A higher connectivity with the Salience network indicates a role in processing relevant stimuli and integrating sensory-motor information [26]. The Left Premotor 6d2 showed strong connectivity with the Sensorimotor network, highlighting its function in motor coordination. Moderate connectivity with the Dorsal Attention and Frontoparietal networks suggests involvement in motor tasks requiring attention and cognitive control. The lower connectivity with the Language, Default Mode, Cerebellar, Visual, and Salience networks highlights its motor control functions.

The Right Premotor 6d3 demonstrated a robust connectivity with the Sensorimotor network, underscoring its pivotal role in executing complex motor tasks [10]. The strong functional connectivity with the Dorsal Attention network could suggest that Right Premotor 6d3 is integral to spatial attention, visually guided movements, task processing, and motor coordination [27]. Additionally, its high connectivity with the Salience network indicates its importance in integrating relevant sensory inputs with motor actions [28]. In contrast, its lower connectivity with the Language, Frontoparietal, Default Mode, Cerebellar, and Visual networks highlights its primary function in motor execution rather than broader cognitive processes [29]. Similarly, the Left Premotor 6d3 exhibited strong connectivity with the Sensorimotor network, affirming its role in motor control. The connectivity with the Dorsal Attention network, mirroring the pattern seen in the Right Premotor 6d3, further supports its involvement in spatial attention and visually guided movements. Its high connectivity with the Salience network suggests a key role in processing salient stimuli for motor coordination. The lower connectivity with the Language, Frontoparietal, Default Mode, Cerebellar, and Visual networks emphasizes the Left Premotor 6d3′s specialized role in motor control, distinct from broader cognitive functions.

The results also revealed that the Right (presma) maintained a robust connectivity with the premotor areas, particularly with right-side regions such as the Right Premotor 6d1. This region showed a moderate connectivity with the Sensorimotor, Dorsal Attention, and Frontoparietal networks, which reflects its role in coordinating motor functions and integrating cognitive tasks [30,31]. The strong connectivity with the Salience network suggests its involvement in prioritizing and managing motor-related sensory information. The low connectivity with the Language, Default Mode, Cerebellar, and Visual networks underscores its primary focus on motor-related tasks [32]. Similarly, the Left presma exhibited a strong connectivity with the left-side premotor regions and moderate connectivity with the Sensorimotor, Dorsal Attention, and Frontoparietal networks. The high connectivity with the Salience network indicates its role in integrating significant sensory inputs for motor execution. This pattern emphasizes its function in linking motor planning with higher-order cognitive processes. The generally low connectivity with the Language, Default Mode, Cerebellar, and Visual networks further highlights its specialization in motor control.

The Right SMA maintained a strong connectivity within the premotor and supplementary motor areas, especially with right-side regions such as Right Premotor 6d1 and Left SMA. It demonstrated a high connectivity with the Sensorimotor network, indicating its role in integrating sensory input with motor execution. Moderate connectivity was observed with the Dorsal Attention and Frontoparietal networks, reflecting its involvement in coordinating motor functions and integrating cognitive tasks. The higher connectivity with the Salience network suggests its role in prioritizing and managing motor-related sensory information [28]. Its connectivity with the Language, Default Mode, Cerebellar, and Visual networks was generally low, underscoring its primary focus on motor-related tasks [32]. The Left SMA exhibited a strong connectivity with the premotor and supplementary motor areas, particularly with left-side regions such as Left Premotor 6d1 and Right SMA. It showed a high connectivity with the Sensorimotor network, emphasizing its role in motor coordination. Moderate connectivity with the Dorsal Attention and Frontoparietal networks highlights its involvement in attentional processes and cognitive control tasks. Similar to the Right SMA, the Left SMA displayed a higher connectivity with the Salience network, indicating its role in integrating significant sensory inputs for motor execution [28]. Connectivity with the Language, Default Mode, Cerebellar, and Visual networks was generally low, indicating its specialization in motor control.

The results of this study showed that all ten regions displayed strong intranetwork connectivity within the premotor and supplementary motor areas, underscoring their integrated role in motor control. However, differences emerged in their connectivity with other networks. The Sensorimotor and Dorsal Attention networks consistently showed a moderate-to-high connectivity across all regions, reflecting their collaborative roles in motor execution and attention. The Salience network exhibited varying degrees of connectivity, indicating its involvement in integrating relevant stimuli with motor actions, but only with specialized sub-regions. In contrast, the lower connectivity with cognitive and sensory networks such as the Language, Default Mode, Cerebellar, and Visual networks underscores the primary motor focus of these regions. These differences in connectivity patterns can be attributed to the functional specialization of each sub-region [33,34,35,36]. Functional connectivity analysis of these sub-regions reveals that the premotor and supplementary motor areas are not only pivotal in motor control but also integrate functions related to attention, cognitive control, and sensory processing, and these are dependent on the sub-region of interest being examined. Additionally, connectivity with the Frontoparietal network indicates their role in higher-order executive functions and cognitive control [22]. The Salience network’s connectivity with these regions highlights their role in processing and prioritizing significant sensory inputs for motor coordination. This investigation is significant because it considers the detailed anatomical and functional subdivisions within the premotor and supplementary motor areas. Understanding these subdivisions and their specific connectivity patterns is vital for interpreting the broader neurophysiological landscape and its implications for both healthy brain function and neurological disorders.

In addition, this study highlights the importance of considering and investigating sub-regions as differences have been detected in the role of each sub-region. This approach aligns with contemporary efforts to unravel the complexities of brain function through detailed, region-specific investigations [33,36,37,38]. Investigating specific sub-regions rather than whole areas is crucial for understanding the nuanced roles these regions play in health and disease. Sub-regional analysis can reveal specific connectivity patterns that might be masked when considering broader regions. For example, distinct connectivity profiles in sub-regions of the premotor cortex can provide insights into specific motor deficits observed in neurological disorders such as Parkinson’s disease [39]. This detailed understanding can inform targeted therapeutic interventions and improve disease management strategies.

### Limitations and Future Directions

This study has several limitations. The age range of the participants (18–30 years) limits the generalizability of the findings to older populations. Additionally, the study’s reliance on right-handed individuals may overlook lateralization differences in left-handed individuals. Future research should consider a more diverse age range and include left-handed participants to explore potential differences in connectivity patterns. Incorporating other tools, such as diffusion tensor imaging (DTI) and magnetoencephalography (MEG), can provide complementary insights into the structural and functional connectivity of these sub-regions. Correlating imaging findings with histological data can validate the observed connectivity patterns and provide a deeper understanding of the underlying neural substrates. Future studies should also incorporate task-based fMRI to explore how these regions interact during specific motor and cognitive tasks. Task-based fMRI can reveal dynamic changes in connectivity and identify task-specific networks that are not apparent during resting-state fMRI. The reliance on open access datasets has potential limitations, such as constraints on manipulating data resolution. Using a smaller kernel for smoothing could enhance the effects of functional connectivity. Future studies should consider these limitations and address them in their investigations. Future studies should aim for larger and more balanced samples, with a detailed examination of handedness effects, to fully explore potential gender-specific differences in functional connectivity. Additionally, incorporating more diverse participant demographics and advanced imaging techniques will enhance the generalizability and depth of the findings. Such studies could provide a more comprehensive understanding of how gender and handedness influence brain connectivity patterns.

## 5. Conclusions

Overall, the ten sub-regions within the premotor and supplementary motor areas demonstrated very strong within-network connectivity, underscoring their coordinated roles in motor control. The connectivity with other networks, including the Language, Frontoparietal, Default Mode, Cerebellar, Visual, Dorsal Attention, Sensorimotor, and Salience networks, ranged from moderate to low. The highest connections were observed with the Sensorimotor, Dorsal Attention, and Salience networks, highlighting the primary involvement of these regions in motor functions. These findings provide valuable insights into the specialized and integrated roles of the premotor and supplementary motor areas in the broader context of brain functional connectivity. Further research, incorporating diverse populations, advanced imaging techniques, and task-based paradigms, will enhance our understanding of these critical brain sub-regions.

## Figures and Tables

**Table 1:** Baseline characteristics.

Characteristic	Total	Females	Males
Number	198	123	75
Age (years)	21.03 (2.31)	21.21 (2.26)	20.72 (2.38)
Neurological history	None	None	None

## Data Availability

The data sample were taken from the Cambridge-Buckner data sample (open access) (http://fcon_1000.projects.nitrc.org (accessed on 9 April 2022)).
